# Current Status and Associated Factors of Depression and Anxiety Among the Chinese Residents During the Period of Low Transmission of COVID-19

**DOI:** 10.3389/fpsyg.2021.700376

**Published:** 2021-09-27

**Authors:** Xin Shen, Shijiao Yan, Hui Cao, Jing Feng, Zihui Lei, Weixin Zhang, Chuanzhu Lv, Yong Gan

**Affiliations:** ^1^Department of Social Medicine and Health Management, School of Public Health, Tongji Medical College, Huazhong University of Science and Technology, Wuhan, China; ^2^School of Public Health, Hainan Medical University, Haikou, China; ^3^Key Laboratory of Emergency and Trauma of Ministry of Education, Hainan Medical University, Haikou, China; ^4^Department of Labor Economics and Management, Beijing Vocational College of Labour and Social Security, Beijing, China; ^5^School of Public Health, Jilin University, Changchun, China; ^6^Emergency Medicine Center, Sichuan Provincial People’s Hospital, University of Electronic Science and Technology of China, Chengdu, China; ^7^Research Unit of Island Emergency Medicine, Chinese Academy of Medical Sciences (No. 2019RU013), Hainan Medical University, Haikou, China

**Keywords:** epidemic, COVID-19, low transmission, risk factors, prevalence, depression, anxiety

## Abstract

**Background:** The outbreak of coronavirus disease 2019 (COVID-19) has contributed to depression and anxiety among the general population in China. The purpose of this study is to investigate the prevalence and associated factors of these psychological problems among Chinese adults during the period of low transmission, which could reflect the long-term depression and anxiety of the COVID-19 outbreak.

**Methods:** A cross-sectional survey was conducted in China from 4 to 26 February 2021. Convenient sampling strategy was adopted to recruit participators. Participants were asked to filled out the questions that assessed questionnaire on the residents’ depression and anxiety.

**Results:** A total of 2,361 residents filled out the questionnaire. The mean age was 29.72 years (*SD* = 6.94) and majority of respondents were female (60.10%). Among the respondents, 421 (17.83%), 1470 (62.26%), and 470 (19.91%) were from eastern, central, and western China, respectively. 1704 (72.17%) consented COVID-19 information has been disclosed timely. 142 (6.01%) and 130 (5.51%) patients suffered from depression and anxiety symptoms. Furthermore, some influencing factors were found, including marital status, place of residence, employment status.

**Conclusion:** This study revealed that anxiety and depression still are potential depression and anxiety for some residents, which suggested early recognition and initiation of interventions during the period of low transmission is still indispensable.

## Introduction

The COVID-19 epidemic was first detected in China at the end of December 2019, when unexplained cases of clustered pneumonia were detected (Nishiura et al., 2020). The Chinese New Year holiday, which coincides with the COVID-19 outbreak, is one of the most festive times of the year in China, causing mass panic when the virus was declared “human-to-human transmission” (Vella et al., 2020). Since the outbreak, the Chinese government has responded quickly, imposing a lockdown and travel restrictions on Wuhan on January 23, an unprecedented move to contain the spread of the epidemic. Within days, the quarantine was extended to other provinces and cities, affecting more than 50 million people in total. Many stayed at home and isolated to prevent infection (Horton, 2020). The constant emergence of infectious diseases caused fear (Xiang et al., 2020). COVID-19 is more contagious and spreads faster than previous outbreaks and pandemics, which could further exacerbate depression and anxiety in the public (Meo et al., 2020). Therefore, timely psychological assessment and appropriate intervention are necessary measures to prevent depressive and anxiety.

The epidemic is a major health crisis affecting several countries with high transmission and mortality rates, which are associated with adverse mental health consequences. Studies have shown that the global population is under extreme strain, leading to a higher risk of anxiety and depression during the COVID-19 outbreak (Özdin and Bayrak, 2020; Wang et al., 2020). Vulnerable populations and health care professionals are particularly affected by the mental health effects of the pandemic (Rajkumar, 2020). A systematic review and meta-analysis found the prevalence of anxiety and depression among health professionals was high during the pandemic (Pappa et al., 2020). The general population is also highly affected by the psychological impact of the COVID-19. Depression and anxiety could reduce patients’ quality of life and increase the risk of chronic physical illness and suicide (Ettman et al., 2020; Rodríguez-Rey et al., 2020; Tan et al., 2020; van der Velden et al., 2020; Goularte et al., 2021). A Chinese study assessed the mental health burden of the COVID-19 pandemic on the general population and revealed that anxiety and depressive were prevalent in 35.1%, and 20.1% of the population, respectively (Huang and Zhao, 2020). Similarly, a study in China by Qiu et al. (2020) revealed that 35% of respondents had psychological distress. Other studies conducted during the COVID-19 period also showed that the prevalence of depression and anxiety was 906 (33%) and 517 (18%), respectively, in Italy (Mazza et al., 2020), 81 (23.6%), and 155 (45.1%) in Turkey (Özdin and Bayrak, 2020). Stress and anxiety further affect the physical and psychological health status (Hu et al., 2020; Liu et al., 2020; Wang et al., 2020) and results in negative health outcomes (Roy-Byrne et al., 2008; Smeeding et al., 2010) such as heart disease, high blood pressure, diabetes. Besides, stress and depression weakens the immune system (Kiecolt-Glaser et al., 2002a,b), and hurt the body’s ability to fight infection (Esterling et al., 1994). Therefore, it is important to understand the depression and anxiety can be alleviated and to consider early intervention (Mahmoudi et al., 2015; Blake et al., 2020).

Unlike traumatic events at the individual level, the COVID-19 outbreak is an ongoing crisis for every member of society. There are profound and widespread psychosocial effects on individuals, communities and people at the international level during outbreaks of infectious diseases. In the early days of the COVID-19 outbreak in 2020, the number of cases in China rose sharply. The number reached eighty thousand in April, with several days of increases of more than a thousand (Cucinotta and Vanelli, 2020; Lau et al., 2020; Wang et al., 2020b). However, after the implementation of control measures, the epidemic was brought under control in China. By January 2021, the total number of cases in China had not exceeded ninety thousand, showing a slow growth (Liu Z. et al., 2021; Lu et al., 2021). Currently, although the epidemic in China has entered a period of low transmission, the prevalence of anxiety and depression in the population of acute infectious diseases is not clear. By investigating the anxiety and depression prevalence of residents in the low transmission period, this study can reflect the long-term impact of COVID-19, and also identify which types of residents are more likely to contain anxiety and depression in the long term, with proposing targeted strategies for mental health protection after the outbreak of similar acute infectious diseases in the future. As more and more countries enter the low transmission period, it become important to investigate the prevalence and risk factors of depression and anxiety in the population at this stage of infectious disease outbreak. 4–26 February, 2021 is considered as the China New Year. We conducted our study during this period and assumed that some of the residents were still suffering from anxiety and depression. Our survey respondents are residents who are older than 18 years old and live in China for more than 12 months and we only surveyed populations. This study explored the anxiety and depression prevalence in the Chinese population at one year after the COVID-19 outbreak, and clarified the potential psychological problems of the residents, which can provide a reference for health care and mental health policy makers.

## Materials and Methods

### Ethics Statement

This study protocol was approved by the institutional review board of Tongji Medical College of Huazhong University of Science and Technology, Wuhan, China. All methods are performed in accordance with relevant guidelines and regulations. Respondents were informed that their participation was voluntary and implied consent on the completion of the questionnaire.

### Study Participants and Survey Design

A cross-sectional survey was conducted in China from 4 to 26 February 2021. Convenient sampling strategy was adopted to recruit participants; the research team used WeChat, China’s most popular social media platform, to publicize and distribute survey links to their network members. Network members were requested to distribute the survey invitation to all their contacts. Respondents were stratified according to the eastern (Beijing, Tianjin, Hebei, Liaoning, Shanghai, Jiangsu, Zhejiang, Fujian, Shandong, Guangdong, and Hainan), central (Shanxi, Jilin, Heilongjiang, Anhui, Jiangxi, Henan, Hubei, and Hunan) and western (Chongqing, Sichuan, Guizhou, Yunnan, Tibet, Shaanxi, Gansu, Qinghai, Ningxia, Xinjiang, Inner Mongolia, and Guangxi) regions of China. Participants were informed that their participation was voluntary and their consent was implied by their completion of the questionnaire. The eligibility criteria are as follows: (1) Chinese citizens aged 18 or above; (2) Ability to understand and read Chinese. Exclusion criteria include: (1) Residents under the age of 18; (2) Residents who have resided in China for less than 12 months.

### Instruments

The survey consisted of questions that assessed (1) demographic background, (2) Center for Epidemiological Studies-Depression (CES-D) scale, and (3) The Self-rating Anxiety Scale (SAS) scale. Demographic information, including gender, age, marital status, place of residence, highest educational level attained, region, employment status and weather participants’ relative or friend has experienced COVID-19 were collected. Whether this psychological problem originates from COVID-19 is mainly due to the fact that this study have emphasized to the respondents in the survey process that the answer to this psychological question needs to be based on the context of COVID-19. Specifically, for each depression and anxiety item, residents need to specify that the various adverse mental states are specifically attributable to COVID-19. In this way, the relationship between psychological problems and COVID-19 is suggested.

CES-D was used to assess depressive symptoms. It includes 20 items; each item has a score of four, ranging from 0 (“little or no time”) to 3 (“most or almost all time”). The total score is 0–60 points, the higher the score, the more severe the depressive symptoms. CES-D classifies participants according to the shard total scores, without rating levels. On the original CES-D scale, a total score of 16 was used to detect the presence of depressive symptoms (Cosco et al., 2020). However, a large number of studies have assessed the diagnostic accuracy of CES-D in detecting depression in the general population and have proposed multiple cutoff points, such as a cutoff point of 18 for elderly people living in residential homes (Dozeman et al., 2011) and a cut-off score of 22 in older Chinese (Cheng and Chan, 2005). A meta-analysis study systematically reviewed 28 CES-D studies, including several Chinese studies, and came up with an optimal cut-off point of 20 points (Vilagut et al., 2016). As a result, an overall score of 20 or higher was considered an indicator of depressive symptoms, consistent with previous research (Jiang et al., 2019). This scale has good reliability and validity, and has been widely used in Chinese population. In this study, the Cronbach’s alpha coefficient of the scale was 0.91.

SAS was used to assess an individual’s level of anxiety (Jegede, 1979; Lindsay and Michie, 1988; Olatunji et al., 2006). There are 20 items in the scale, with 15 forward scores and 5 reverse scores, which is a 4-point score. The cumulative score for each item is multiplied by 1.25 to get the standard total score. A total score of <50 was classified as no anxiety, 50–59 as mild anxiety, 60–69 as moderate anxiety, and ≥70 as severe anxiety. In this study, the Cronbach’s alpha coefficient of the scale was 0.92.

### Statistical Methods

Descriptive analysis includes the mean and standard deviation of continuous variables and the quantity and percentage of classified data. No clustering was observed in the respondents (correlation = 0.03, *P* < 0.001). Therefore, the multivariable linear regression analysis model was used to estimate factors associated with anxiety and depression in residents. We used a variance inflation factor to assess multicollinearity. All analyses were carried out using STATA 12.0, and all differences were tested using two-tailed tests and a *P*-value of 0.05 was considered statistically significant.

## Results

### Descriptive Statistics

A total of 2,453 residents received the questionnaire. The response rate was 96.24% with 21 participants not responding and 71 questionnaires not completed. The remaining 2,361 complete questionnaires were used in our analysis.

Table 1 reports the socio-demographic characteristics of the 2,361 respondents. The mean age was 29.72 years (*SD* = 6.94) and majority of respondents were female (60.10%). Among the respondents, 421 (17.83%), 1470 (62.26%), and 470 (19.91%) were from eastern, central, and western China, respectively. Most respondents (89.24%) report having attained a bachelor’s degree or higher. More than half of the participants were unemployed (57.05%), unmarried (66.07%), and lived in urban (58.11%). Many of the participants were students, so they were mostly unmarried and unemployed. The mean CES-D scores and SAS Scores of respondents was 8.96 (*SD* = 10.78) and 28.94 (*SD* = 10.79). Using a cutoff score of an overall score of 20 or higher was considered an indicator of depressive for CES-D and more than 50 scores was the indicator of anxiety for SAS. With this average, it is possible to conclude that the sample does not have anxiety or depression. More precisely, 142 (6.01%) and 130 (5.51%) patients had probable suffer from depression and anxiety.

**TABLE 1:** Statistical description of study samples.

Variables	N (%)
Total	2361 (100)
**Gender**	
Male	942 (39.90)
Female	1419 (61.10)
**Age group, y**	
18–44	1845 (78.14)
45–59	369 (15.63)
>60	111 (4.70)
**Marital status**	
Unmarried	1560 (66.07)
Married	801 (33.93)
**Place of residence**	
Urban	1372 (58.11)
Rural	989 (41.89)
**Highest educational level**	
Primary school or below	68 (2.88)
middle school	186 (7.88)
College degree or above	2107 (89.24)
**Region**	
Eastern China	421 (17.83)
Central China	1470 (62.26)
Western China	470 (19.91)
**Employment status**	
Employed	1014 (42.95)
Unemployed	1347 (57.05)
**Relative or friend has experienced COVID-19**	
Yes	206 (8.73)
No	2155 (91.27)

Tables 2, 3 listed the multivariable linear regression analysis results of depression and anxiety factors in respondents. Data distribution coincidence the assumptions of normality and homogeneity of the variances. VIF value were 2.26 (depression) and 2.09 (anxiety), respectively. Residents over 60 years of age (β = 2.533, 198 95%CI: 1.103 ∼ 3.964) had higher CES-D scores. And residents over 60 years of age (β = 4.437, 95%CI: 1.478 ∼ 7.397), lived in rural (β = 1.573, 95%CI: 0.021 ∼ 3.166) and had relative or friend has experienced COVID-19 (β = 2.481, 95%CI: 0.056 ∼ 5.018) had higher SAS scores; while residents were married (β = –2.929, 95%CI: –4.975 ∼ –0.883) had lower scores. *R*^2^ value were 23.2% (depression) and 36.3% (anxiety), respectively.

**TABLE 2:** Regression analysis of associated factors for CES-D Scores among respondents.

Variables	Unstandardized coefficients		Standardized coefficients	*t*	*p*	95%CI
		
	β	*SE*	β			
Total	37.812	1.168	NA	32.378	<0.001	35.523 ∼ 40.101
**Gender (Ref: Male)**						
Female	–0.065	0.356	–0.004	–0.182	0.856	–0.763 ∼ 0.634
**Age group, y (Ref: 18–44)**						
45–59	0.714	0.483	0.031	1.478	0.139	–0.233 ∼ 1.661
>60	2.533	0.730	0.073	3.472	0.001	1.103 ∼ 3.964
**Marital status (Ref: Unmarried)**						
Married	–0.446	0.504	–0.025	–0.884	0.377	–1.435 ∼ 0.543
**Place of residence (Ref: Urban)**						
Rural	0.185	0.393	0.011	0.470	0.638	–0.585 ∼ 0.955
**Highest educational level attained (Ref: Primary school or below)**						
Middle school	–2.117	1.224	–0.068	–1.729	0.084	–4.516 ∼ 0.282
College degree or above	–1.404	1.076	–0.052	–1.305	0.192	–3.513 ∼ 0.704
**Region (Ref: Eastern China)**						
Central China	0.311	0.479	0.018	0.648	0.517	–0.629 ∼ 1.250
Western China	0.604	0.569	0.029	1.060	0.289	–0.512 ∼ 1.719
**Employment status (Ref: Employed)**						
Unemployed	0.627	0.494	0.037	1.270	0.204	–0.340 ∼ 1.595
**Relative or friend has experienced COVID-19 (Ref: No)**						
Yes	0.111	0.625	0.004	0.177	0.859	–1.115 ∼ 1.337
Ref: Reference						

**TABLE 3:** Regression analysis of associated factors for SAS Scores among respondents.

Variables	Unstandardized coefficients		Standardized coefficients	*t*	*p*	95%CI
		
	β	*SE*	β			
Total	73.355	2.417	NA	30.354	<0.001	68.619 ∼ 78.092
**Gender (Ref: Male)**						
Female	0.235	0.738	0.007	0.319	0.750	–1.210 ∼ 1.681
**Age group, y (Ref: 18–44)**						
45–59	–0.337	1.000	–0.007	–0.337	0.736	–2.297 ∼ 1.623
>60	4.437	1.510	0.061	2.939	0.003	1.478 ∼ 7.397
**Marital status (Ref: Unmarried)**						
Married	–2.929	1.044	–0.079	–2.806	0.005	–4.975 ∼ –0.883
**Place of residence (Ref: Urban)**						
Rural	1.573	0.813	0.044	2.134	0.043	0.021 ∼ 3.166
**Highest educational level attained (Ref: Primary school or below)**						
Middle school	–1.997	2.533	–0.031	–0.788	0.431	–6.962 ∼ 2.968
College degree or above	0.288	2.226	0.005	0.129	0.897	–4.076 ∼ 4.651
**Region (Ref: Eastern China)**						
Central China	0.391	0.992	0.011	0.394	0.694	–1.554 ∼ 2.335
Western China	2.071	1.178	0.047	1.758	0.079	–0.238 ∼ 4.380
**Employment status (Ref: Employed)**						
Unemployed	1.220	1.021	0.035	1.194	0.232	–0.782 ∼ 3.222
**Relative or friend has experienced COVID-19 (Ref: No)**						
Yes	2.481	1.294	0.040	2.317	0.035	0.056 ∼ 5.018
Ref: Reference						

## Discussion

The COVID-19 outbreak has disrupted people’s normal lives. Cases of COVID-19 have increased rapidly around the world, causing feelings of uncertainty, depression, and anxiety. Moreover, the implementation of quarantine measures could also have a psychological impact on the residents. The research of Emerson (2020) showed that social distance has a significant impact on loneliness and health behaviors among American adults. In reality, previous studies have shown that at the beginning of the COVID-19 epidemic in China, the prevalence of anxiety and depression in the public was 28.8 and 16.5%, respectively (Wang et al., 2020). This cross-sectional study, based on 2,361 participants, assessed the prevalence and risk factors of depression and anxiety in the general Chinese population during periods of low transmission. We found that 142 (6.01%) and 130 (5.51%) patients suffered from depression and anxiety symptoms. In addition, age, marital status, location of residence, whether a relative or friend had COVID-19, and employment status were factors that influenced anxiety and depression. Because of the urgent need to control the spread of this epidemic, one of WHO’s main recommendations is to implement social distancing procedures, which involve minimizing social and physical contact between people, making it impossible for older people to participate in various social activities, which may increase the risk of psychological problems (Williams et al., 2020).

Research by Shah et al. (2021) has shown that people who are unemployed are more likely to suffer from stress and depression during COVID-19; Tee et al. (2020) found that unmarried people were more likely to experience stress, anxiety and depression during the pandemic.

Previous research has explained this phenomenon that due to adaptive mechanisms that lack the capacity to deal with crises, which can be used to manage stress associated with the current pandemic (Goularte et al., 2021). Our findings also showed a higher prevalence of anxiety among unemployed and unmarried people. It can be speculated that this community is more prone to negative emotions in the crisis due to the lack of stable career and family support (Pasco et al., 2008; Hu et al., 2018; Gloster et al., 2020). Another group worth discussing is the participants who live in rural areas. Liu L. et al. (2021) showed that during COVID-19, there were significant differences in the mental states of urban and rural residents in China. People who live in rural areas are more likely to suffer from anxiety, depression, and other mental problem, which is consistent with our results. A systematic review by Wang et al. (2020a) also reported similar findings. One possible explanation is that rural residents are more at risk of COVID-19 and more likely to report psychological problems due to poor economic development and poor medical care in rural areas (Liu et al., 2014; Liu and Mao, 2019). Therefore, more attention should be paid to protecting the mental health of these populations during periods of low transmission. Our study also showed that individuals with a relative or friend who experienced COVID-19 were more likely to report anxiety symptoms, similar to previous studies (Wang et al., 2020). The possible explanation for this phenomenon is that they were more aware of COVID-19 infectivity, and therefore more fearful. Therefore, more attention should be paid to protecting the mental health of these populations during periods of low transmission.

During periods of low transmission, some people still have psychological problems such as anxiety, depression, and stress (Kang et al., 2020; Liem et al., 2020; Xiang et al., 2020). The purpose of this study was to describe the prevalence of anxiety and depression, two major psychological problems, in different populations, and to analyze the potential risk factors during periods of low transmission. This could prompt government agencies and psychologists to pay attention to people’s mental health. In addition, the interpretation of the conclusion requires special attention. Although the results of this study identified some potentially vulnerable groups, the results should be treated with caution. In reality, although these people showed higher values in the CES-D and SAS scales, the cut-off points do not indicate depression nor anxiety, because the averages are below the 50 points (SAS) and 20 points (CES-D). Therefore, further research is needed to explore this topic in the future.

## Limitations

This is the first study to measure depression and anxiety in the population in the low transmission period. We used a nationwide sample of the Chinese population, and the results could be useful for countries entering a phase of low transmission. However, there were also some limitations. First, we use a snowball sampling strategy. Snowball sampling is a sampling method of selecting potential interviewes based on existing interviewes, rather than random sampling. The university’s admissions information is posted on the website, which leads to the majority of respondents being young students. Therefore, the selection of participants in our study was biased, and the sample population of the study may not be a good representative of the actual patterns of the general population. In addition, the great disparity in size of some subsamples (e.g., age, relative or friend infected with COVID) may increase the probability of making a type 1 error, so consequently, new controlled studies are necessary to verify the results obtained in this research. Second, our study was cross-sectional; we cannot infer a causal relationship between risk factors and depressive symptoms. Therefore, a cohort study is needed to verify this temporal relationship. Third, CES-D is only a screening tool, not a diagnostic tool, although it is already widely used and validated in China. Fourth, this study used a self-administered online questionnaire, so participants needed to be able to use online tools, which might affect their responses to the questionnaire. In addition, future research should also explore more potential factors affecting depression and anxiety of residents, such as specific occupation, working in a home office and shift work.

## Conclusion

This study indicates that during the low transmission period in China, there were still some general population suffered from depression and anxiety symptoms. People who were older, unemployed, unmarried, live in rural areas, and have a relative or friend who has experienced COVID-19 are more likely to have depression and anxiety. Based on our findings, we recommend the establishment of targeted psychological interventions to improve the mental health of the public during low transmission periods.

## Data Availability Statement

The raw data supporting the conclusions of this article will be made available by the authors, without undue reservation.

## Ethics Statement

This study protocol was approved by the institutional review board of Tongji Medical College of Huazhong University of Science and Technology, Wuhan, China. The patients/participants provided their written informed consent to participate in this study.

## Author Contributions

XS, SY, and YG conceived and designed the study. JF and ZL participated in the acquisition of data. XS and SY analyzed the data. HC and WZ gave advice on methodology. XS and SY drafted the manuscript. XS, SY, CL, and YG revised the manuscript. All authors read and approved the final manuscript. YG is the guarantor of this work and had full access to all the data in the study and takes responsibility for its integrity and the accuracy of the data analysis.

## Conflict of Interest

The authors declare that the research was conducted in the absence of any commercial or financial relationships that could be construed as a potential conflict of interest.

## Publisher’s Note

All claims expressed in this article are solely those of the authors and do not necessarily represent those of their affiliated organizations, or those of the publisher, the editors and the reviewers. Any product that may be evaluated in this article, or claim that may be made by its manufacturer, is not guaranteed or endorsed by the publisher.
